# Supplementary figures and images for: Accelerometer-assessed outdoor physical activity is associated with meteorological conditions among older adults: Cross-sectional results from the OUTDOOR ACTIVE study
Source: PLoS One. 2020 Jan 24;15(1):e0228053. doi: 10.1371/journal.pone.0228053 (PMC6980536; doi:10.1371/journal.pone.0228053)

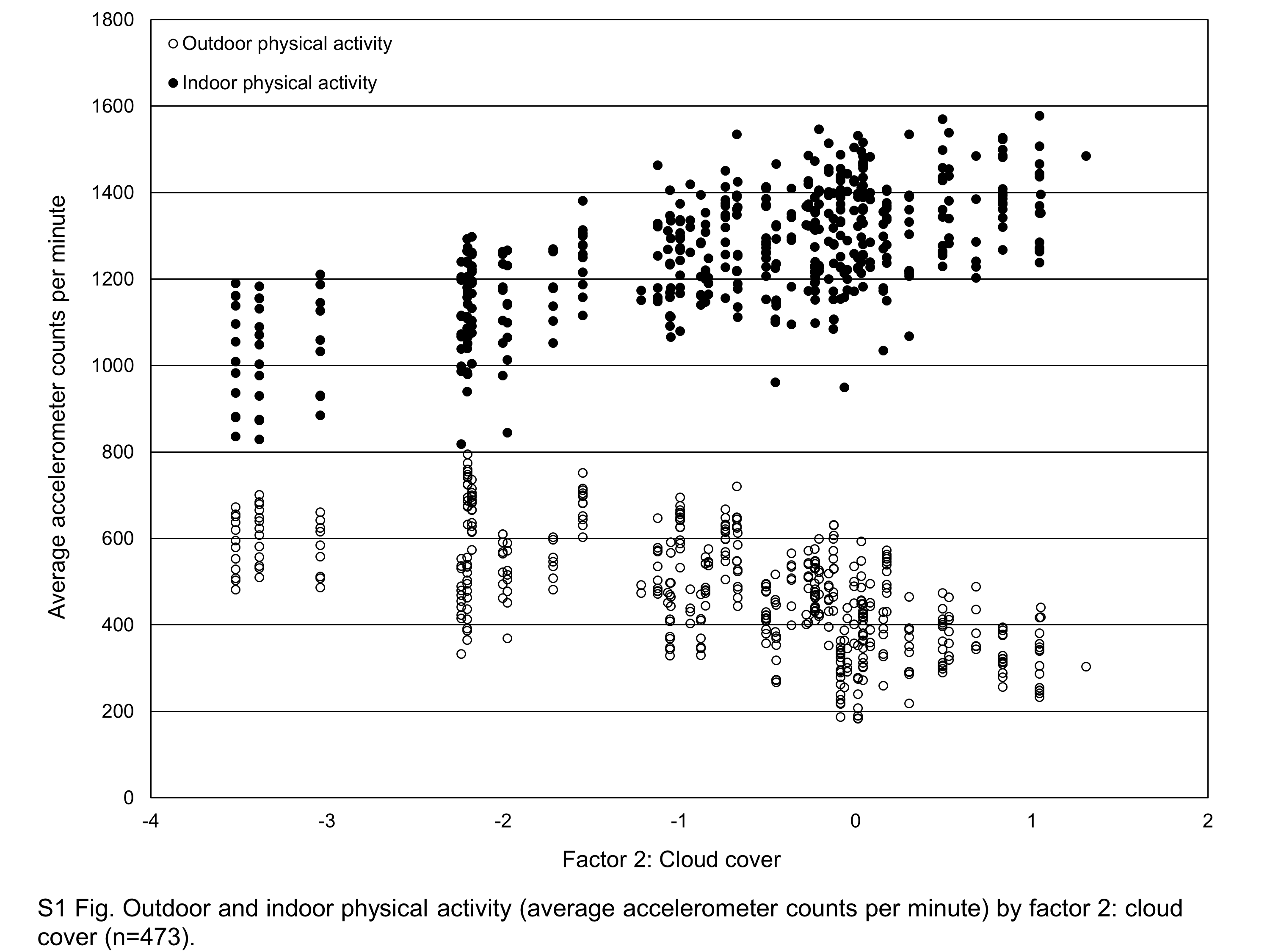

Supplement: S1 Fig — (TIF) [file pone.0228053.s001.tif]
