# Supplementary material for: Accelerometer-assessed outdoor physical activity is associated with meteorological conditions among older adults: Cross-sectional results from the OUTDOOR ACTIVE study
Source: PLoS One. 2020 Jan 24;15(1):e0228053. doi: 10.1371/journal.pone.0228053 (PMC6980536; doi:10.1371/journal.pone.0228053)
Supplement: S1 Table — (PDF) [file pone.0228053.s002.pdf]

S1 Table. Pearson correlation coefficients between all meteorological variables.

		1	2	3	4	5	6	7	8	9	10	11	12	13	14
1	Mean temperature (°C)	**1.00**													
2	Minimum temperature at 2 m (°C)	**0.95**	**1.00**												
3	Minimum temperature at 5 cm (°C)	**0.93**	**0.99**	**1.00**											
4	Mean vapor pressure (hPa)	**0.93**	**0.93**	**0.93**	**1.00**										
5	Maximum temperature at 2 m (°C)	**0.98**	**0.89**	**0.86**	**0.88**	**1.00**									
6	Day length (h)	**0.69**	**0.58**	**0.56**	**0.60**	**0.76**	**1.00**								
7	Sunshine (h)	**0.39**	**0.19**	**0.15**	**0.17**	**0.52**	**0.53**	**1.00**							
8	Mean relative humidity (%)	**-0.43**	**-0.28**	**-0.24**	**-0.12**	**-0.52**	**-0.53**	**-0.76**	**1.00**						
9	Mean cloud cover (1/8)	-0.04	**0.12**	**0.18**	**0.12**	**-0.16**	**-0.21**	**-0.74**	**0.51**	**1.00**					
10	Mean wind speed (km/h)	-0.09	-0.01	0.02	**-0.16**	**-0.16**	**-0.32**	**-0.22**	-0.09	**0.15**	**1.00**				
11	Maximum wind speed (km/h)	0.06	0.11	**0.13**	0.02	0.02	**-0.14**	-0.11	-0.10	0.08	**0.85**	**1.00**			
12	Mean air pressure (hPa)	0.07	0.02	-0.01	0.04	0.09	-0.02	**0.22**	-0.07	**-0.24**	**-0.30**	**-0.32**	**1.00**		
13	Snow depth (cm)	**-0.37**	**-0.36**	**-0.33**	**-0.28**	**-0.36**	**-0.22**	**-0.14**	0.09	**0.12**	0.07	-0.02	**-0.16**	**1.00**	
14	Precipitation (mm)	0.03	0.09	**0.12**	**0.13**	-0.01	-0.02	**-0.22**	**0.24**	**0.26**	**0.20**	**0.30**	**-0.31**	-0.04	**1.00**

Statistically significant correlation coefficients ($p < 0.05$) are shown in bold characters
