# Supplementary material for: Accelerometer-assessed outdoor physical activity is associated with meteorological conditions among older adults: Cross-sectional results from the OUTDOOR ACTIVE study
Source: PLoS One. 2020 Jan 24;15(1):e0228053. doi: 10.1371/journal.pone.0228053 (PMC6980536; doi:10.1371/journal.pone.0228053)
Supplement: S2 Table — (PDF) [file pone.0228053.s003.pdf]

S2 Table. Association of OPA (average accelerometer CPM) and meteorological factors with OPA defined as lux ≥ 500.

	OPA (unadjusted)		OPA (adjusted)	
	Women (n=68, 238 days)	Men (n=60, 235 days)	Women (n=68, 238 days)	Men (n=60, 235 days)
	β (95%-CL)	β (95%-CL)	β (95%-CL)	β (95%-CL)
Factor 1 Temperature	133.4 (49.9, 216.9)**	146.4 (52.5, 240.2)**	139.0 (56.6, 221.5)**	143.6 (49.4, 237.8)**
Factor 2 Cloud cover	-129.8 (-171.7, -87.9)***	-113.8 (-161.4, -66.3)***	-134.5 (-176.4, -92.7)***	-114.4 (-161.9, -66.8)***
Factor 3 Wind	-90.1 (-153.3, -26.9)**	-80.6 (-154.4, -6.8)*	-101.2 (-164.0, -38.4)**	-77.8 (-151.8, -3.8)*
Factor 4 No precipitation	3.7 (-79.0, 86.4)	-21.2 (-112.8, 70.4)	-5.3 (-86.7, 76.0)	-23.3 (-115.1, 68.6)
Age (years)			-15.1 (-25.2, -5.0)**	-11.6 (-24.7, 1.5)
Body-mass-index (kg/m^2)			-9.1 (-17.2, -1.1)*	-0.1 (-10.2, 10.0)

* *p*-value < 0.05, ** *p*-value < 0.01, *** *p*-value < 0.001

OPA: Outdoor physical activity

CPM: Counts per minute

CL: Confidence limits

Linear mixed models (random factors: days, study subjects), stratified by sex, unadjusted and adjusted for age and body-mass-index.

Meteorological factors were derived by principal component analysis.

Includes only days with a maximum temperature ≥ 20°C.
