# Supplementary material for: Accelerometer-assessed outdoor physical activity is associated with meteorological conditions among older adults: Cross-sectional results from the OUTDOOR ACTIVE study
Source: PLoS One. 2020 Jan 24;15(1):e0228053. doi: 10.1371/journal.pone.0228053 (PMC6980536; doi:10.1371/journal.pone.0228053)
Supplement: S3 Table — (PDF) [file pone.0228053.s004.pdf]

S3 Table. Association of IPA (average accelerometer CPM) and meteorological factors with IPA defined as lux < 500.

	IPA (unadjusted)		IPA (adjusted)	
	Women (n=68, 238 days)	Men (n=60, 235 days)	Women (n=68, 238 days)	Men (n=60, 235 days)
	β (95%-CL)	β (95%-CL)	β (95%-CL)	β (95%-CL)
Factor 1 Temperature	112.3 (-40.1, 264.7)	79.0 (-34.5, 192.5)	113.2 (-31.5, 257.9)	81.7 (-32.2, 195.5)
Factor 2 Cloud cover	81.0 (4.5, 157.4)*	67.6 (10.1, 125.1)*	79.6 (6.1, 153.0)	65.3 (7.8, 122.7)*
Factor 3 Wind	48.7 (-66.6, 164.1)	-96.9 (-186.2, -7.7)*	30.8 (-79.4, 141.0)	-97.8 (-187.2, -8.4)*
Factor 4 No precipitation	245.7 (94.7, 396.7)**	-3.9 (-114.7, 106.9)	212.9 (70.1, 355.7)**	-1.2 (-112.1, 109.8)
Age (years)			-48.8 (-66.5, -31.1)***	-13.4 (-29.3, 2.5)
Body-mass-index (kg/m^2)			-16.0 (-30.2, -1.9)*	-5.8 (-18.0, 6.4)

* *p*-value < 0.05, ** *p*-value < 0.01, *** *p*-value < 0.001

IPA: Indoor physical activity

CPM: Counts per minute

CL: Confidence limits

Linear mixed models (random factors: days, study subjects), stratified by sex, unadjusted and adjusted for age and body-mass-index.

Meteorological factors were derived by principal component analysis.

Includes only days with a maximum temperature ≥ 20°C.
